# Dosimetrically administered nebulized morphine for breathlessness in very severe chronic obstructive pulmonary disease: a randomized, controlled trial

**DOI:** 10.1186/s12890-017-0535-y

**Published:** 2017-12-11

**Authors:** Piotr Janowiak, Małgorzata Krajnik, Zygmunt Podolec, Tomasz Bandurski, Iwona Damps-Konstańska, Piotr Sobański, David C. Currow, Ewa Jassem

**Affiliations:** 1 Department of Pneumonology and Allergology, Medical University of Gdańsk, Dębinki 7, 80-211 Gdańsk, Poland; 2 Department of Palliative Care, Nicolaus Copernicus University, Collegium Medicum in Bydgoszcz, M. Curie Skłodowskiej 9, 85-094 Bydgoszcz, Poland; 3 Department of Aerosology and Aerosol Bioengineering, Research and Development Centre of MEDiNET, Juliusza Lea 114, 30-133 Kraków, Poland; 4 Department of Radiology Informatics and Statistics, Medical University of Gdańsk, Tuwima 15, 80-210 Gdańsk, Poland; 5 Palliativzentrum Hildegard, Sankt Alban-Ring 151, 4020 Basel, Switzerland; 6 Gynaecological Cancer Center, University Hospital Basel, Spitalstrasse 21, 4031 Basel, Switzerland; 7 Faculty of Health, University of Technology Sydney, PO Box 123, Broadway, NSW 2007 Australia

## Abstract

**Background:**

Systemic morphine has evidence to support its use for reducing breathlessness in patients with severe chronic obstructive pulmonary disease (COPD). The effectiveness of the nebulized route, however, has not yet been confirmed. Recent studies have shown that opioid receptors are localized within epithelium of human trachea and large bronchi, a target site for a dosimetric nebulizer. The aim of this study was to compare any clinical or statistical differences in breathlessness intensity between nebulized 2.0% morphine and 0,9% NaCl in patients with very severe COPD.

**Methods:**

The study was a double-blind, controlled, cross-over trial. Participants received morphine or NaCl during two 4-day periods. Sequence of periods was randomized. The primary outcome measure was reduction of breathlessness intensity now by ≥20 mm using a 100 mm visual analogue scale (VAS) at baseline, 15, 30, 60, 120, 180 and 240 min after daily administration, during normal activities.

**Results:**

Ten of 11 patients included completed the study protocol. All patients experienced clinically and statistically significant (*p* < 0.0001) breathlessness reduction during morphine nebulization. Mean VAS changes for morphine and 0.9% NaCl periods were 25.4 mm (standard deviation (SD): 9.0; median: 23,0; range: 14.0 to 41,5; confidence interval (CI): 95%) and 6.3 mm (SD: 7.8; median: 6.8; range: −11,5 to 19,5; CI: 95%), respectively. No treatment emergent adverse effects were noted.

**Discussion:**

Our study showed superiority of dosimetrically administered nebulized morphine compared to NaCl in reducing breathlessness. This may have been achieved through morphine’s direct action on receptors in large airways, although a systemic effect from absorption through the lungs cannot be excluded.

**Trial registration:**

Retrospectively registered (07.03.2017), ISRCTN14865597

## Background

Chronic obstructive pulmonary disease (COPD) is the second, most frequent chronic respiratory entity [1]. In the last year of life of patients with severe and very severe COPD, breathlessness occurs in up to 98% [2]. There is evidence for the use of systemic, oral or parenteral, opioids to reduce the symptom of chronic breathlessness, in particular morphine [3]. Although there have been sporadic reports on central respiratory depression after systemic use of this compound, appropriately administered morphine is considered a relatively safe medication [3]. Nevertheless, an alternative route of delivery by nebulization was proposed to reduce other burdensome side effects of systemic morphine, such as constipation or dizziness. Rationale for this approach was supported by both in vitro and in vivo studies which showed beneficial effects of opioids delivered directly to the bronchial tree [4]. Hitherto, the effectiveness of nebulized morphine in breathlessness was demonstrated only in a few uncontrolled studies and case reports [5–7]. Notably, beneficial effect of nebulized morphine was not confirmed in two systematic reviews [8, 9], whereas the most recent review showed a modest benefit [10], resulting from just one positive randomized trial [11], with low to moderate evidence across the available trials. Hence, in contrast to systemic delivery, nebulized morphine has not been considered a standard treatment.

Recent studies have shown that opioid receptors are localized within epithelium of human trachea and large bronchi on unmyelinated C nerve fibers and pulmonary neuroendocrine cells (PNEC) [12]. It was proposed that morphine, acting directly on PNECs and C-fibers, might limit neurogenic inflammation and afferent signal propagation to central nervous system, decreasing the sensation of breathlessness [12]. Hence, nebulization might be a clinically effective route of morphine administration provided the drug particles reach the large bronchi. The standard nebulizers, however, perform inhalation poorly, as a large majority of the dose is lost, while the remaining dose is dispersed in the bronchial tree in an unpredictable manner. Recently, we demonstrated that the large bronchi could be easily targeted by a dosimetrically operated nebulizer [13].

The aim of the present study was to compare clinical effectiveness of morphine and 0,9% NaCl, both delivered by the same inhalation system, calibrated to target large airways, in patients with very severe COPD and chronic breathlessness in a double blind randomized study. The null hypothesis was that there was no clinically and statistically significant difference in breathlessness between nebulized morphine and 0,9% NaCl. The primary endpoint was the intensity of breathlessness measured by visual analogue scale (VAS) with daily activity and the secondary endpoints were the most effective dose of morphine, exercise tolerance measured by Wilcock’s test and treatment safety.

## Methods

### Subjects

Subjects were recruited from the Pomeranian Model of Integrated Care for Patients With Severe COPD led by the Department of Allergology and Pneumonology at the University Clinical Centre, Gdańsk, Poland. Recruitment took place from 04.03.2014 to 01.03.2016. A total of 270 patients were screened, 30 of whom met the following inclusion criteria: (a) age above 50 years; (b) diagnosis of COPD group D, according to 2013 Global Initiative For Chronic Obstructive Lung Disease (GOLD) guidelines [14] (considering most recent FEV1% values from spirometry, performed in stable state) which are consistent with their 2017 version [15]; (c) stage IV airflow limitation i.e. FEV1% < 30%, according to 2011 GOLD classification [16]; (d) breathlessness rated 3 or 4 in the modified Medical Research Council scale (mMRC) breathlessness scale [17]; (e) current non-smoker; (f) written informed consent. Exclusion criteria included: (a) other coexisting severe chronic lung diseases, such as lung cancer; (a) breathlessness caused by other than COPD chronic diseases, such as heart failure or renal failure; (c) inability to give informed consent; (d) previous history of respiratory depression after opioid administration or allergic reactions to opioids; (e) ongoing opioid treatment for any indication; and (f) COPD exacerbation within the last month.

### Protocol

The study was a randomized, double-blind, controlled, dose increment, cross-over trial, uniform within sequences and periods. Patients with persistent breathlessness were hospitalized for 8 days in the stable period of the disease. Patients were administered two consecutive nebulization periods, each lasting 4 days: 4 once-daily doses of 0,9% NaCl nebulization and 4 once-daily doses of 2% morphine hydrochloride water solution nebulization or vice versa. The sequence of periods was determined using online software for simple randomization: Research Randomizer ver. 3.0 [18]. The randomization was performed by the independent hospital pharmacist and the dispensing of the study medications was blinded. Both substances (4 ml’s of solution) were delivered by dosimetric nebulizer (PNEUMONEB®) equipped with BCTS-S head (Bronchial Control Treatment System – Sidestream) comprising both nebulizer and pneumotachometer. Constant analyses of patients’ breathing patterns by the pneumotachometer allowed controlled introduction of a drug bolus into inspired air, during the third quarter of the inspiration. Dosimetric nebulizer was calibrated with static spirometry values, allowing for individualization of the procedure.

**Fig. 1:**
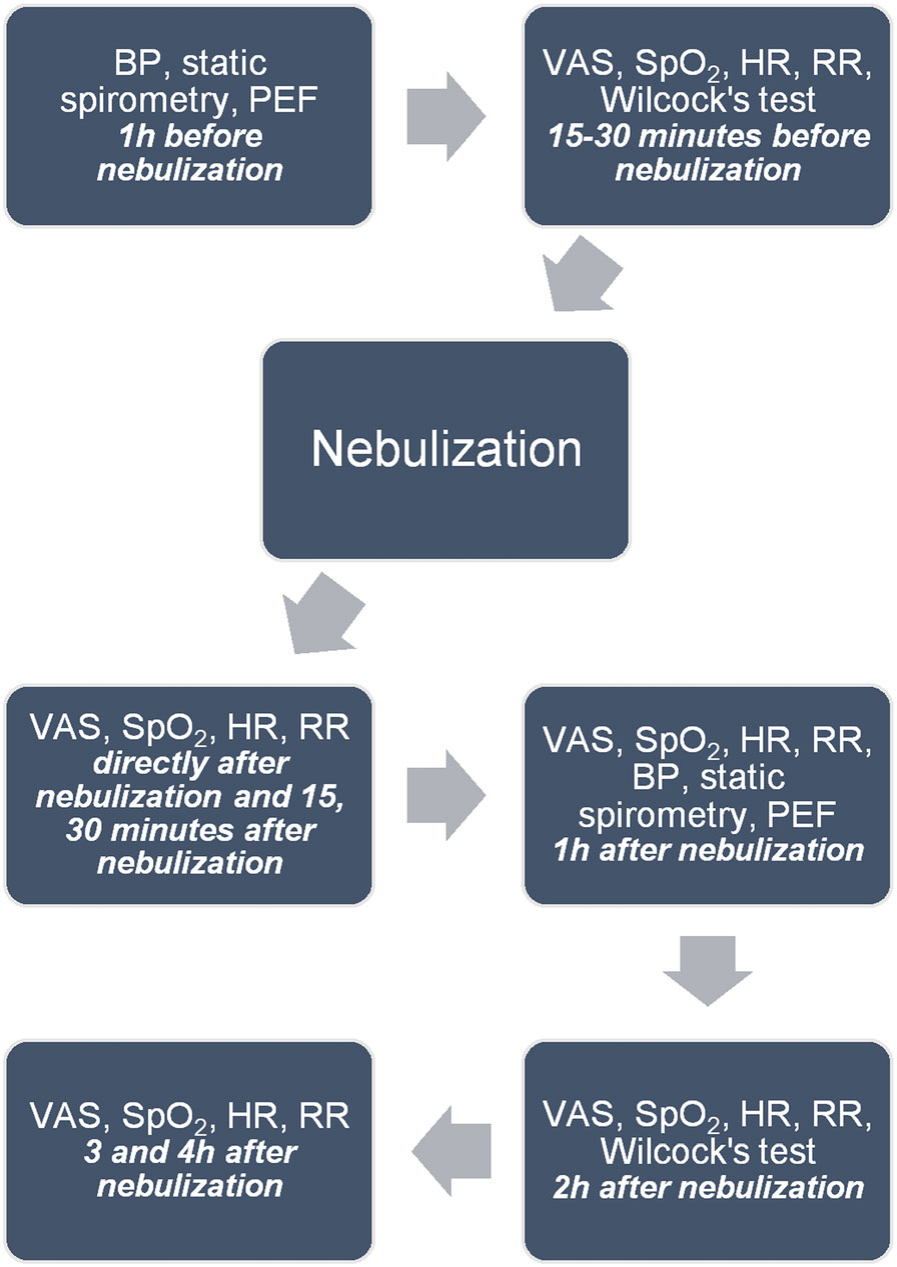
Trial protocol. BP, blood pressure; PEF, peak expiratory flow; VAS, visual analogue scale; SpO_2_, peripheral capillary oxygen saturation; HR, heart rate; RR, respiratory rate

Treatment efficacy was assessed by breathlessness intensity now measured during normal, daily activity on a 100 mm, horizontal visual analogue scale (VAS), anchored at one end with a sad face and a happy face at the other. Exercise tolerance was measured by Wilcock’s test [19] Fig. 1. VAS is simple to use, has high sensitivity and reproducibility, and was validated in measuring breathlessness intensity changes [20]. Patients were asked to estimate their breathlessness on VAS at several time points – 15-30 min before the nebulization, immediately after the nebulization and 15 min, 30 min, 1, 2, 3 and 4 h after the nebulization. Heart rate, respiratory rate and peripheral capillary oxygen saturation (SpO_2_) were measured at the same time points Fig. 1. Following nebulization, patients were encouraged to engage in their routine activities to monitor potential changes in perceived breathlessness. During Wilcock’s test, considered a practical means to measure exercise tolerance in patients with severe breathlessness, participants were asked to read numbers, as quickly and clearly as they could from a page with a grid of numbers. The procedure was repeated five times and the highest number of numbers read and the number read per breath were recorded [19]. Number reading test was performed 15–30 min before and 2 h after nebulization.

Both substances were delivered once daily in a titrated manner until the clinically significant response (the reduction of breathlessness by more than 20 mm in VAS [21, 22]) was reached or substantial side effects occurred. In order to detect this drop we were comparing the best and the worst VAS scores across the whole 4 day period. Morphine doses for 4 consecutive days were: 1, 2, 3 and 5 mg.

To further ensure treatment safety, patients underwent static spirometry and peak expiratory flow (PEF) measurement: both 1 h before and 1 h after the nebulization Fig. 1. Blood pressure was measured before each spirometry. Vital capacity (VC), inspiratory vital capacity (IVC) and inspiratory capacity (IC) were recorded. In patients older than 70 years, IC reference values were calculated from the equation provided by Lisboa et al. [23]. The drop in the spirometric values by more than 15 percentage points and PEF by more than 20 l/min was considered clinically important [24, 25]. Due to the natural variability of lung function and, consequently, spirometry results, we decided to compare the worst and the best test values separately.

### Statistical analysis

Statistical analysis was performed using STATISTICA® version 12 software (StatSoft Inc., OK, USA, 1984–2014) by an independent statistician, on data acquired on patients who received at least one morphine nebulization. VAS results for NaCl and morphine treatment were compared day-to-day and minute-to-minute using repeated measures analysis of variance. Day-to-day analysis was calculated using VAS scores gathered before nebulization and in 240 min after. Post-hoc analysis was performed with Scheffe test and Bonferroni correction. Mean VAS change and mean Wilcock’s test’s changes were calculated for each one of four periods separately, from differences between best and worst test values obtained across each 4 day period, and then compared using a two-tailed dependent t-test. A *p*-value of less than 0.05 was considered statistically significant.

### Sample size calculation

Sample size was calculated using the following formula:

$$ n=\frac{{\left({z}_{\alpha }+{z}_{\beta}\right)}^2\times {\sigma}^2\ }{2\times {\left(\mu -{\mu}_0-\delta \right)}^2} $$

where z = standard score, α = 0.05 (probability of a type 1 error), β = 0.85 (probability of a type 2 error), δ = 20 (superiority margin), μ-μ_0_ = 40 (acceptable mean difference) and σ^2^ = 484 (population variance) which resulted in 5 patients per sequence. Population variance was based on the data provided by Johnson et al. [26] in their study on populations with chronic refractory breathlessness.

Study protocol was approved by the Independent Bioethics Committee for Research of Medical University of Gdansk (NKBBN/269/2012) and financed by the internal university grant no. ST-553.

## Results

Out of the 30 patients primary screened for the study, 5 declined participation, and 8 died before entering the trial. Due to the observed, bigger than expected, differences in VAS scores between the two study arms [27], the trial needed to be stopped, ethically, after 10 of 11 admitted patients completed study protocol Table 1 i.e. after reaching calculated sample size. One patient developed infective COPD exacerbation on the second day of trial during 0.9% NaCl nebulization phase and was excluded from the analysis. Two patients were included into the study in spite of higher values of FEV1% (35,5% and 31% – as assessed, consecutively, 3 and 5 months before the study). At the time of screening they were unable to perform dynamic spirometry due to the steady progress of the disease. Standard COPD treatment remained unchanged during the study and each patient received maximum COPD treatment.

**Table 1:** Patient characteristics

	Sex	FEV1 (%)	Age (yrs)	BMI	LTOT	Comorbidities	Drug sequence
1	M	35.5	59	31	–	HT, CAD, DM, LPR	NaCl, morphine
2	M	29.2	73	14	–	PAF, Anemia	Morphine, NaCl
3	M	29.7	59	23	–	HT	Morphine, NaCl
4	M	22.2	63	32	Yes	HT, DM	Morphine, NaCl
5	M	24.2	83	16	–	HT, CAD	Morphine, NaCl
6	M	28.7	62	26	–	TR, MR, OSA	NaCl, morphine
7	M	17.4	60	25	–	–	NaCl, morphine
8	M	28.6	74	23	–	–	NaCl, morphine
9	F	28.0	72	27	Yes	HT	Morphine, NaCl
10	F	31.0	67	30	Yes	GERD	NaCl, morphine
*Mean*	–	*27.5%*	*67.2*	*24.7*	–	–	–

FEV1, forced expiratory volume in one second; BMI, body mass index; LTOT, long term oxygen therapy; M, male; F, female; HT, hypertension; CAD, coronary artery disease; DM, diabetes mellitus; LPR, laryngo-pharyngeal reflux; PAF, paroxysmal atrial fibrillation; TR, tricuspid regurgitation; MR, mitral regurgitation; OSA, obstructive sleep apnea; GERD, gastroesophageal reflux disease.

**Fig. 2:**
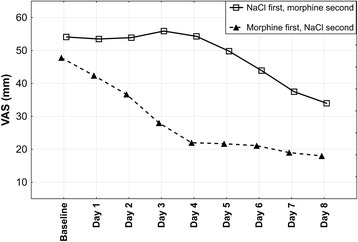
Mean visual analogue scale breathlessness scores for Groups 1 and 2. VAS, visual analogue scale; baseline, breathlessness assessment made on the first day of treatment, before nebulization. *Mean visual analogue scale breathlessness scores for 8 days were calculated from visual analogue scale scores taken in fourth hour after nebulization*

**Fig. 3:**
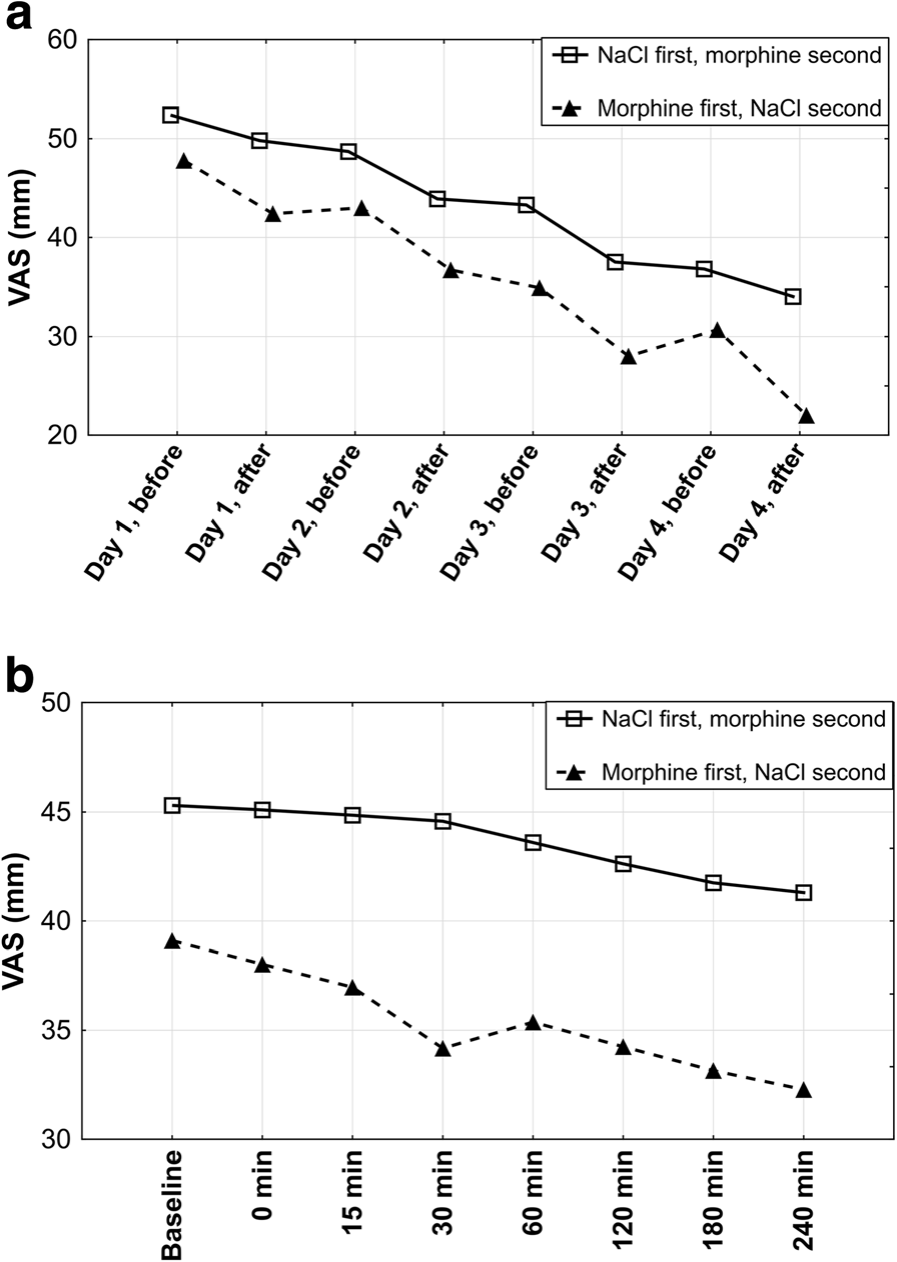
Changes in visual analogue scale breathlessness scores during morphine nebulization. Section A: daily changes in visual analogue scale (VAS) during morphine nebulization; ‘*before*’ and ‘*after*’ refer to the moment of morphine nebulization. Section B: minute changes in VAS after morphine nebulization; ‘*baseline*’ refers to the assessment made on the first day of treatment, before nebulization

Mean VAS changes for morphine and 0.9% NaCl periods were 25.4 mm (standard deviation (SD): 9.0; median: 23,0; range: 14.0 to 41,5; confidence interval (CI): 95%) and 6.3 mm (SD: 7.8; median: 6.8; range: −11,5 to 19,5; CI: 95%), respectively. In both groups (either starting with morphine or 0.9% NaCl) breathlessness gradually decreased during morphine phase (*p* < 0.0001 for both: day-to-day and minute-to-minute analysis) and a statistically significant breathlessness reduction was already achieved on the second day of the period (*p* < 0.002) Figs. 2 and 3. Morphine dose, however, was raised further to achieve 20 mm VAS drop. 7 out of 10 patients required raising morphine dose to 3 mg on day 3, remaining 3 patients met aforementioned clinical criterion on day 4 at a dose of 5 mg. Statistically significant and sustained decrease in breathlessness during morphine nebulization started in thirtieth minute (*p* = 0.005) after nebulization and peaked four hours after nebulization Fig. 3. All study patients expressed their willingness to continue morphine nebulization at home.

**Fig. 4:**
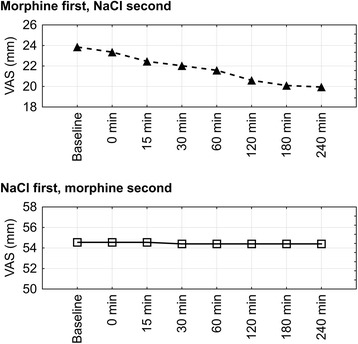
Changes in visual analogue scale breathlessness scores during 0,9% NaCl nebulization. VAS, visual analogue scale; baseline, breathlessness assessment made on the first day of treatment, before nebulization

During NaCl nebulization, a statistically significant (*p* < 0.0001 for day-to-day analysis and *p* < 0.00001 for minute-to-minute analysis) drop in breathlessness intensity started on the third day (*p* = 0.04) in those patients who started their NaCl nebulization after four days of morphine nebulization. However, this drop (mean VAS change of 9.8 mm) did not meet a preset clinical significant cut-off of 20 mm. This improvement was not seen in Group 1 (*p* = 0.926 for day-to-day analysis and *p* = 0.908 for minute-to-minute analysis), where NaCl nebulization preceded morphine nebulization Fig. 4. The mechanism for this change is not immediately apparent.

**Fig. 5:**
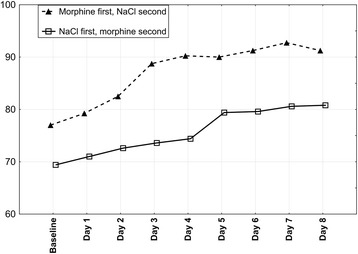
Wilcock’s test: number of numbers read. Baseline, Wilcock’s test performed on the first day of treatment, before nebulization. *Test results for days 1–8 were taken after nebulization*

A significant improvement (*p* < 0.05) in Wilcock’s test, independent of the substance used, was observed in both groups Fig. 5 with the exception of number read per breath during NaCl nebulization (*p* = 0.06). We did not detect statistically significant difference between influence of 0.9% NaCl and morphine on the test. The mean “number of numbers read” increase was 12.7 for the morphine and 8.1 in NaCl period (*p* = 0,09), respectively, whereas mean “number read per breath” change was 4.3 and 3.7 (*p* = 0,28), respectively. Aforementioned data were obtained for 9 (“number of numbers read”) and 8 (“number read per breath”) patients. One patient, due to visual impairment, could not perform Wilcock’s test properly and in another collection of necessary “number read per breath” was impossible due to severe breathlessness.

**Fig. 6:**
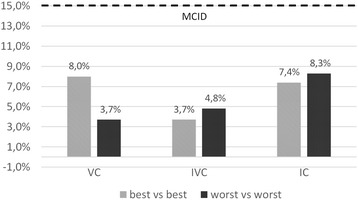
Changes in spirometry values, expressed as percentage points; comparison of best and worst values obtained during morphine and 0,9% NaCl periods. MCID, Minimal Clinically Important Difference [24]; VC, Vital Capacity; IVC, Inspiratory Vital Capacity; IC, Inspiratory Capacity

**Fig. 7:**
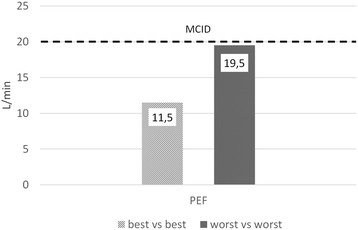
Changes in peak expiratory flow values, expressed as L/min; comparison of best and worst values obtained during morphine and 0,9% NaCl periods. MCID, Minimal Clinically Important Difference [25]; PEF, Peak Expiratory Flow

Morphine nebulization was well tolerated and there was no significant side effects, apart from a bitter taste (8 patients) and transient, mild dizziness immediately after the nebulization (2 patients). Mean respiratory rate during NaCl period equaled 20.2 breaths/min, during morphine period – 20.18 (*p* = 0.74). No changes in heart rate, blood pressure and SpO_2_ and no significant decrease in spirometric parameters was observed between periods Figs. 6 and 7.

## Discussion

Our study showed a reduction of chronic breathlessness accompanying severe COPD by dosimetric nebulization system. We were able to demonstrate in the randomized setting that inhaled morphine at a dose 3–5 mg decreases breathlessness by more than 20 mm in the VAS, with minimal side effects and that this improvement is sustained for at least 24 h after 1 dose. We were able to meet this high threshold despite the evidence that VAS change greater than 10 mm might already be clinically significant in chronic breathlessness [26]. Nonetheless, we adopted 20 mm in view of earlier, unsuccessful nebulized morphine trials. Despite positive VAS results we did not detect significant difference in Wilcock’s test. Taking into consideration that Wilcock’s test results correlate with FVC [19] this can be explained by the lack of clinically significant changes in results of static spirometry. An apparent limitation of this study was inability to achieve effective blinding, since this requirement could not be accomplished due to a strong bitter taste of nebulized morphine. Moreover, trial design changed after publication of study by Johnson et al. [26] which allowed us to properly calculate sample size.

Our positive results differ most likely due to the differences in the methods of nebulization. Virtually all previous studies used opioids delivered by jet nebulizers widely known for their unreliable drug delivery, or did not specify the equipment used explicitly. Indeed, up to 70% of the drug delivered by jet nebulizer is deposited inside the apparatus and up to 20% is lost into the environment leaving barely 10% of set dose that reaches the lungs [28]. Moreover, aerosol is produced in a constant fashion, resulting in a considerable variability in drug deposition among consecutive nebulizations delivered by the same jet nebulizer [28]. Furthermore, only one group [29] among the randomised controlled studies analyzed by systematic reviews [8–10] chose mass median aerodynamic diameter (MMAD) suitable for deposition in the large airways (3,1–4,9 μm). The aforementioned might explain why despite using a wide range of nebulized morphine doses (1 mg–50 mg [30, 31]) previous researchers were mostly unable to achieve positive results.

This study used dosimetric nebulizer (PNEUMONEB®) coupled with BCTS-S head. PNEUMONEB® analyzes patients breathing pattern in a real time and delivers drug aerosol bolus in the third quarter of the inspiration. This approach minimizes drug losses to the environment and to the inner surface of the device [28], increases drug deposition in the trachea and large bronchi [32], where PNECs and C-fibers are located, and ensures the repeatability between consecutive drug deliveries [33]. BCTS-S head produces large aerosol particles with MMAD of 4.6 μm [13], further increasing morphine deposition in the trachea and large bronchi [32]. This method may increase drug deposition in lungs up to 60% of the dose [13], of which a significant portion reaches opioid receptors. It is worth underlining that pharmacokinetics of nebulized morphine delivered by PNEUMONEB® in cancer patients [34] is substantially different from that of morphine delivered by other routes [35–39]. Although bioavailability of nebulized morphine was estimated in earlier, jet nebulizer studies at less than 10% [40, 41], its effective doses in our study (3 and 5 mg) were close to ones given parenterally [42]. However, it is worth mentioning that plasma levels of morphine and its metabolites after nebulization with PNEUMONEB® with BCTS-S head [34] are lower than after intravenous [36] delivery or after nebulization with AERx® [43], a system designed to deliver drugs systemically through alveoli.

Further work is then required, using PNEUMONEB®, correlated simultaneously with plasma levels of morphine and its metabolites, in order to understand where, centrally or locally, the opioid is having its dominant effect.

The role of dynamic hyperinflation was not considered during this study, however respiratory rates and measured lung volumes remained stable throughout the study. Its contribution to breathlessness needs to be considered in future studies.

### Limitations

This study used standard measures for the assessment of breathlessness but not for any toxicities or harms, relying instead on self-report. Plasma samples were not taken to quantify systemic absorption, and this will be important in future work. Understanding any impact on dynamic hyperinflation will also be important in future studies. Given the findings, a longer washout period would also be justified in future work. The study was registered retrospectively.

## Conclusions

Our study showed an apparent reduction of chronic breathlessness intensity now accompanying severe COPD with morphine delivered by dosimetric nebulization system which ensured delivery of drug to the desired level of the airways. Treatment was effective and safe in all participating patients. In consequence, all of them expressed willingness to continue morphine nebulizations at home. In the majority of patients the effective dose was 3 mg. This positive effect was most probably achieved through direct morphine action on its receptors located in the epithelium of the trachea and large bronchi.

## Abbreviations

BCTS-S: Bronchial Control Treatment System – Sidestream
BMI: body mass index
BP: blood pressure
CAD: coronary artery disease
CI: confidence interval
COPD: chronic obstructive pulmonary disease
DM: diabetes mellitus
F: female
FEV1: forced expiratory volume in one second
GERD: gastroesophageal reflux disease
GOLD: Global Initiative For Chronic Obstructive Lung Disease
HR: heart rate
HT: hypertension
IC: inspiratory capacity
IVC: inspiratory vital capacity
LPR: laryngo-pharyngeal reflux
LTOT: long term oxygen therapy
M: male
MCID: minimal clinically important difference
MMAD: mass median aerodynamic diameter
mMRC: modified Medical Research Council (scale)
MR: mitral regurgitation
OSA: obstructive sleep apnea
PAF: paroxysmal atrial fibrillation
PEF: peak expiratory flow
PNEC: pulmonary neuroendocrine cells
RR: respiratory rate
SD: standard deviation
SpO_2_: peripheral capillary oxygen saturation
TR: tricuspid regurgitation
VAS: visual analogue scale
VC: vital capacity
α: probability of a type 1 error
β: probability of a type 2 error
δ: superiority margin
μ-μ_0_: acceptable mean difference
σ^2^: population variance
